# Seizure Control of Current Shunt on Rats with Temporal Lobe Epilepsy and Neocortical Epilepsy

**DOI:** 10.1371/journal.pone.0086477

**Published:** 2014-01-30

**Authors:** Limin Zhang, Shuli Liang, Guojun Zhang, Zhizhong Liu, Hong Lv, Fang Fang, Yajie Wang, Shaohui Zhang, Xixiong Kang

**Affiliations:** 1 Laboratory Diagnosis Center, Beijing Tiantan Hospital, Capital Medical University, Beijing, China; 2 Department of Neurosurgery, Capital Epilepsy Therapy Center, First Affiliated Hospital of Chinese People's Liberation Army General Hospital, Beijing, China; Beijing Institute of Radiation Medicine, China

## Abstract

**Purpose:**

To examine the effects of current shunt on rats with temporal lobe epilepsy and neocortex epilepsy.

**Experimental Design:**

A kainic acid (KA)-induced model of temporal lobe seizure and a penicillin-induced model of neocortical partial seizure were used in this study. Rats of each model were randomly allocated into two groups: control and model groups. The model group was further divided into the KA or penicillin group, sham conduction group and conduction group. The current shunt was realized through the implantation of a customized conduction electrode. After surgery, electroencephalogram (EEG) was recorded for two hours for each rat under anesthesia. Subsequently, the rats were video monitored for 72 h to detect the occurrence of behavioral seizures upon awakening. The average number and duration of seizures on EEG and the number of behavioral seizures were measured.

**Results:**

In KA model, the number of total EEG seizures in conduction group (9.57±2.46) was significantly less than that in sham conduction group (15.13±3.45) (*p*<0.01). The duration of EEG seizures in conduction group (26.13±7.81 s) was significantly shorter than that in sham conduction group (34.17±7.25 s) (*p* = 0.001). A significant reduction of behavioral seizures was observed in the conduction group compared with KA (p = 0.000) and sham conduction groups (p = 0.000). In penicillin model, there was a 61% reduction in total EEG seizures in conduction group compared with sham conduction group (p<0.01), and the duration of EEG seizures in conduction group (6.29±2.64 s) was significantly shorter than that in the sham conduction group (12.07±3.81 s) (p = 0.002). A significant reduction of behavioral seizures was observed in conduction group compared with penicillin (p<0.01) and sham conduction groups (p<0.01).

**Conclusion:**

Current shunt effectively reduces the onset and severity of seizures. Current shunt therapy could be an effective alternative minimally invasive approach for temporal lobe epilepsy and neocortex epilepsy.

## Introduction

Despite many alternatives to antiepileptic drugs, 30% of patients with epilepsy remain poorly controlled [1], [2]. Approximately 60% of these patients are candidates for resective surgery [3]. For patients who are not suitable for resective surgery, neuromodulation therapy, including vagus nerve and deep brain stimulation, might provide additional benefits in combination with medical treatment to reduce seizure frequency [4], [5]. But there are still a significant proportion of epilepsy patients for which no adequate therapy is currently available; thus, novel therapeutic strategies are required to treat these patients.

**Figure 1:**
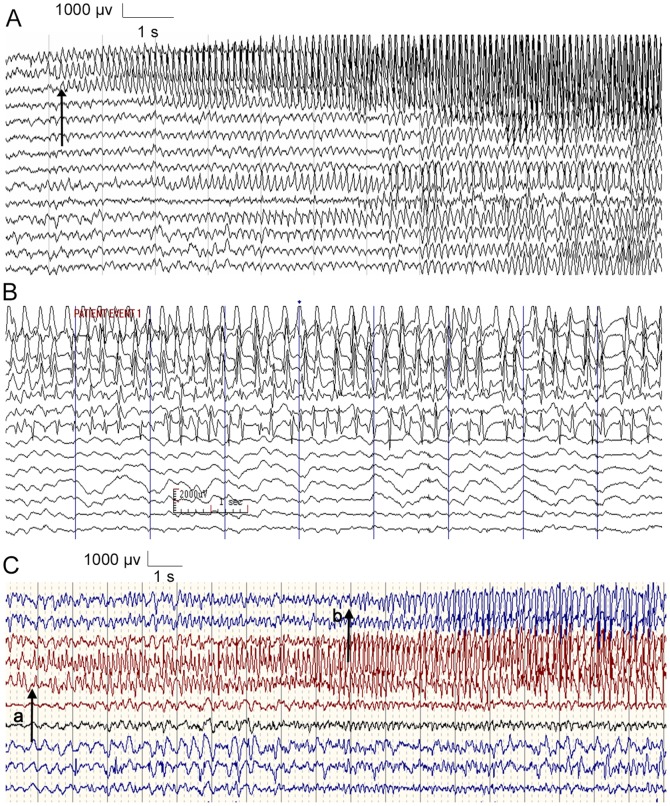
The characteristics of partial seizures on intracranial electrode EEG. (A) The localized original site (arrow); (B) The relatively high voltage of epileptiform discharge; (C) The relatively long propagation time. The spreading time of the discharge from point “a” to point “b” (arrows) was about 9 seconds.

The prevailing view is that seizures are paroxysmal event reflecting abnormal excessive or synchronous neuronal activity in the brain [6]. Studies [7]–[10] on scalp electroencephalogram (EEG) and intracranial electrode EEG have shown that focal seizure activity is typically initiated in a fixed and localized region of the cortex, which subsequently spreads to neighboring regions or more distant areas (Figure 1A); at the initial onset of a seizure, the epileptiform discharge will generate a relatively high voltage (2000∼3000 µV) in intracranial electrode EEG (Figure 1B), with a wave amplitude approximately 30 times that of the scalp EEG (about 50∼100 µV) [7]. In previous experiments, we have also observed this intracranial electrode EEG recording phenomenon. In addition, the propagation of bursting activity in patients with focal epilepsy, from the seizure focus to early spread of ictal discharge, typically has a relatively fixed spreading pathway, and seizure propagation requires one to several seconds, which is much longer than the spreading time of normal nervous electric activity [11], [12](Figure 1C). Thus, the relatively high-voltage ictal epileptic discharge is limited in the fixed onset zone for several seconds during the intracranial EEG recording in patients with focal epilepsy. Furthermore, the current transmits from regions with relatively high voltage towards other regions with low voltage. Based on these findings, we propose that seizures can be controlled through a current shunt using a conduction microelectrode to conduct the epileptic discharge with a relatively high voltage in the seizure focus outside the brain.

This idea has been supported through evidence from previous studies. Brain stimulation has been proposed as an alternative therapy for patients with medically refractory focal seizures who are not candidates for surgical resection. Clinical trials on vagus nerve stimulation have demonstrated that 20∼40% of patients achieve greater than 50% reduction in seizure frequency in the first year of use [13]. The modern era of deep brain stimulation began in the late 1980s with the pioneering work of Benabid and colleagues [14], [15]. These authors showed that chronic high-frequency stimulation results in clinical benefits analogous to those achieved through surgical lesioning, thereby transforming the use of functional neurosurgery for the treatment of movement disorders [16]. Several other sites have since been studied, including the cerebellum, subcortical tissue, thalamus, caudate nucleus, through direct stimulation of the epileptic focus. Studies [17]–[20] on anterior nuclei of thalamus for patients with focal epilepsy showed 46% to 67% reduction of seizures. In addition, Trevelyan [21] showed that seizure propagation could be opposed through feedforward inhibition. Taken together, these results suggest that external factors can influence the intrinsic excitability of neurons.

The current therapies for epilepsy, including drugs, resective surgery, neuromodulation and so on, focus on inhibiting abnormal excessive or synchronous neuronal activity to control seizures; thus the basic mechanism underlying these therapies is “inhibition”. In contrast, we propose a “conduction” mechanism, whereby a current shunt with conduction electrode is used to control seizures. The aim of the present study was to confirm whether current shunt treatment presents anti-epileptic effects in an acute kainic acid (KA)-induced model of temporal lobe seizure and a penicillin-induced model of neocortical partial seizure in rats.

## Materials and Methods

### Ethics Statement

All animal procedures were performed in accordance with the recommendations of the Guide for the Care and Use of Laboratory Animals of the National Institutes of Health. The protocol was approved through the Animal Ethics Board of Capital Medical University Affiliated Beijing Tiantan Hospital. All surgeries were performed under anesthesia using chloral hydrate, and all efforts were made to minimize suffering.

### Animals

Adult male Sprague-Dawley (SD) rats (The Laboratory Animal Center, Academy of Military Medical Science) weighing 220∼260 g were enrolled in this study. The rats were housed two per cage under controlled conditions (20°C∼23°C, 12-h light/12-h dark cycle and 50% relative humidity), with a normal diet of tap water and rat chow ad libitum.

### Part I: KA Induced Temporal Lobe Epilepsy (TLE) Model

#### Animal group and KA model establishment

**Figure 2:**
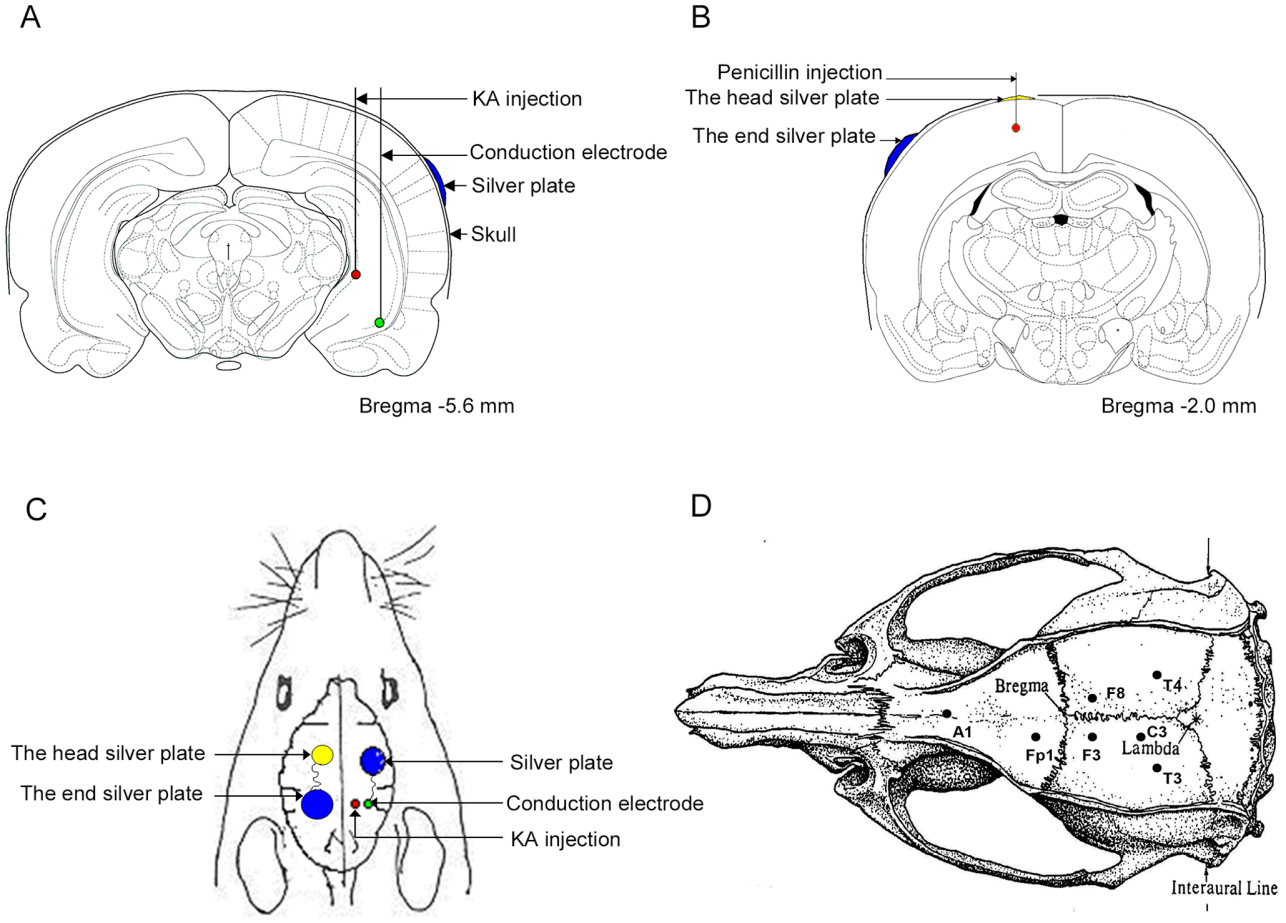
The position of drug injection and electrodes implantation. (A) and (C): Red, KA injection site, and the tip of the recording electrode for the CA3 region of the right hippocampus; Green, the tip of the conduction electrode; and Blue, the end of the conduction electrode, a silver plate. (B) and (C): Red, penicillin injection site; Yellow, the head end of conduction electrode; and Blue, the tail end of conduction electrode. (D) Diagram of the placement of implanted recording electrodes. The EEG signals were recorded from F4, T4, and T3 electrodes in KA rats, and Fp1, F3 and C3 electrodes in penicillin rats against the reference electrode A1 (adapted from Paxinos & Watson, 1997 [19]).

One hundred rats were anesthetized with 10% chloral hydrate (3 ml/kg) intraperitoneally. This anesthetic exhibited a relatively wide safety range and had no obvious inhibitory effect on the respiration of rats. Moreover, the use of chloral hydrate had no obvious effect on the outcomes observed in the present study. The rats were randomly divided into two groups: control group (n = 25, injected with saline) and model group (n = 75, injected with KA). The model group was further divided into three subgroups: KA group (n = 25), sham conduction group (n = 25) and conduction group (n = 25). After the anesthesia was administered, the state of consciousness was regularly assessed through reaction to a toe pinch stimulus. The rats were attached to a stereotactic animal frame (David Kopf Instruments, USA) at the cranial level and a midline incision was made along the scalp to expose the skull. After holes were drilled into the skull, stainless steel screw electrodes were placed epidurally in the frontal sinus and occipital bone as reference and ground electrodes, respectively. The other three intracranial needle recording electrodes were implanted according to Paxinos and Watson’s stereotactic atlas (Figure 2) [22]: the CA3 regions of the right and left hippocampus (T4 and T3), 5.6 mm posterior to bregma, 4.5 mm lateral to midline on both sides, 5.5 mm below the skull; the anterior temporal lobe cortex (F4), 2.0 mm posterior to bregma, 1.5 mm lateral to midline on the right side, 2.2 mm below the skull. All electrodes except T4 were implanted and the connected wires were fixed. We recorded EEG in T3 and F4. Before electrode T4 was implanted and connected with a wire, KA (0.6 µg/0.3 µl, Sigma-Aldrich, St. Louis, MO, USA) was injected at T4 for 5 min using a 0.5 µl Hamilton microsyringe in the rats of the model group. The needle remained in place for 5 min after injection. The T4 electrode was fixed in place. The rats in the control group were subjected to the same surgical procedures, except for injection with 0.3 µl of saline.

#### EEG recording

The electric activity was recorded and analyzed using the Ceegraph Vision Analysis System (Biologic Company, 2008). The following recording parameters were used: 0.3–70 Hz low and high frequency filter and 30 mm/sec recording speed. EEG seizures were recognized against background according to their large amplitude (more than three times baseline amplitude) and high-frequency EEG activity (≥5 Hz) for at least two seconds in the T4 electrode [23]. Focal EEG seizure was defined as the limitation of the ictal discharge on the T4 electrode, and spreading EEG seizure was defined as the ictal discharge spread from the T4 to the T3 or F4 electrode.

#### Conduction electrode implantation

The customized needle conduction electrode comprised a silver needle head end (diameter 0.3 mm) and a tail end silver patch (diameter 5 mm) with both sides naked. Silver silk, for insulation and lagging, was used to connect the head and the tail end. The model was considered successful when EEG seizures were observed at least three times in each rat after KA treatment. Subsequently, a conduction electrode was implanted into the sham conduction group and conduction group during the following four steps. First, a steelhead silicone guide cannula (diameter 0.5 mm) was inserted into the right side of the CA1 region of the hippocampus via a burr hole. The coordinates were AP = 5.6 mm, R = 5.2 mm and V = 7.5 mm (Figure 2). Second, a Teflon-insulated silver needle conduction electrode was inserted into the target region through the guide cannula by an electrode manipulator. Third, the guide cannula and the needle head end of the conduction electrode were fixed onto the skull using dental resin. Last, the remainder of the conduction electrode, with a silver patch tail end, was embedded under the parietal scalp. Approximately 2 mm of the head end of the needle conduction electrode used in the conduction group was naked, while that in the sham conduction group was insulated. EEGs were recorded for two hours after the conduction electrode implantation.

### Part II: Penicillin Induced Neocortical Epilepsy Model

#### Animal group and penicillin model establishment

Sixty rats were randomly divided into two groups: control group (n = 15, injected with saline) and experiment group (n = 45, injected with penicillin). The experiment group was further divided into three groups: penicillin group (n = 15), sham conduction group (n = 15) and conduction group (n = 15). After anesthetizing intraperitoneally with 10% chloral hydrate (3 ml/kg), the rats were attached to a stereotactic animal frame, and a midline incision was made along the scalp to expose the skull. After holes were drilled into the skull, three stainless steel screw recording electrodes (diameter 0.3 mm) were placed epidurally at 2 mm lateral to the midline on left side: one electrode was placed over the frontal cortex, 2 mm anterior to bregma (Fp1); another electrode was placed over the parietal cortex, 2 mm posterior to bregma (F3); and the final electrode was placed over the parietal cortex, 5 mm posterior to bregma (C3) (Figure 2). The reference electrode (A1) was placed epidurally at 7 mm anterior to bregma at the midline. All electrodes, except F3, were implanted and connected with wires. An area near F3, with a 2.5 mm radius, was exposed after removing the skull. Before the electrode F3 was implanted and connected with wire, penicillin (200 IU/µl) was injected 2.2 mm below the skull at 0.2 µl/min using a microsyringe. The needle remained in place for 5 min after injection.

#### EEG recording

EEG seizures were recognized against background according to their large amplitude (more than three times baseline amplitude) and high-frequency EEG activity (≥5 Hz) for at least two seconds in the F3 electrode [23]. The focal EEG seizure was defined as the limitation of the ictal discharge on the F3 electrode, and the spreading EEG seizure was defined as the ictal discharge spread from the F3 to the Fp1 or C3 electrode.

#### Conduction electrode implantation

The patch-conducting electrode comprised a head end silver patch (diameter 4 mm) with one insulated side and one naked side and a tail end silver patch (diameter 5 mm) with both sides naked. Silver silk, for insulation and lagging, connected the two patches. The model was considered as successful when EEG seizures were observed at least three times in each rat after penicillin treatment. Subsequently, a patch conduction electrode was implanted into the rats of sham conduction group and conduction group in the following manner: the head end silver patch was placed on the surface of the exposed cortex near the F3 electrode above the penicillin injection site with the naked side down and fixed using EC adhesive, and the tail end of a silver plate and the connecting silver silk were embedded under the parietal scalp. The head end platinum patch of the conduction electrode used in sham conduction group was insulated on both sides. EEGs were recorded for two hours after conduction electrode implantation.

### Behavioral Observations in the KA and Penicillin Models

The rats in both models were placed in individual cages and continuously video monitored for 3 days following EEG recording after awaking from anesthesia. In the TLE model, 3 rats in the KA group and 2 rats in the sham conduction group died from serious convulsions during monitoring. Thus, we added another 3 rats to the KA group and another 2 rats to the sham conduction group to replace the dead rats, guaranteeing a total of 25 rats in each group. The data obtained from the dead rats were rejected during analysis. A modified scale based on that of Racine [24] was used to score the convulsions: 0.5, jaw clonus; 1, myoclonic jerks of the contralateral forelimb; 2, mild bilateral forelimb clonus; 3, severe bilateral forelimb clonus, lasting for at least 15 s; 4, rearing with back fully extended in addition to severe forelimb clonus; and 5, rearing and falling in addition to severe forelimb clonus. Two trained technicians (F.F. and Z.S.), blinded to the treatments and dates, analyzed the videotapes of the rats for each grade seizure independently, and the average data from two technicians were used as the number of seizures. We grouped the behavioral seizures as minor seizures (Racine 0.5∼2 grades) and serious seizures (Racine 3∼5 grades).

### Confirmation of KA Injection Foci in TLE Model and Rat Sacrifice

After the behavioral observations, 8 rats in TLE models were randomly selected and perfused with 0.1 M phosphate-buffered saline (PBS), followed by transcardial perfusion with 4% paraformaldehyde, under deep anesthesia. The brains were removed and placed in a 10% formalin solution for at least 24 h, followed by paraffin-embedding. Coronal slices (4∼6 µm) containing the hippocampus and electrode tracts were cut using a microtome (RM2155; Leica, Nussloch, Germany), mounted on glass slides, and processed through HE staining to confirm the precise position of KA injection using light microscopy. The remaining rats were euthanized by sodium pentobarbital overdose (1 ml/kg, i.p.) at 3 and 7 d after EEG recording, followed by the rapid extraction of the brain on ice. The hippocampus of TLE rats and the neocortex of neocortical epilepsy rats were rapidly dissected and stored in liquid nitrogen until further study.

### Statistical Analysis

All data were analyzed using SPSS version 18.0 (SPSS Inc., USA). The data were expressed as the means ± standard deviation (SD). Differences between the groups were determined using one-way analysis of variance (ANOVA), followed by Bonferroni post hoc test. A value of *P*<0.05 was considered statistically significant.

## Results

A total of 70 out of the 75 rats in the KA injection group were successful, with 24 rats in the KA group, and 23 rats in sham conduction and conduction groups. A total of 41 out of the 45 rats in the penicillin injection group were successful, with 13 rats in the penicillin group, and 14 rats in sham conduction and conduction groups.

### Part I: KA Induced TLE Model

#### Number of EEG seizures

**Figure 3:**
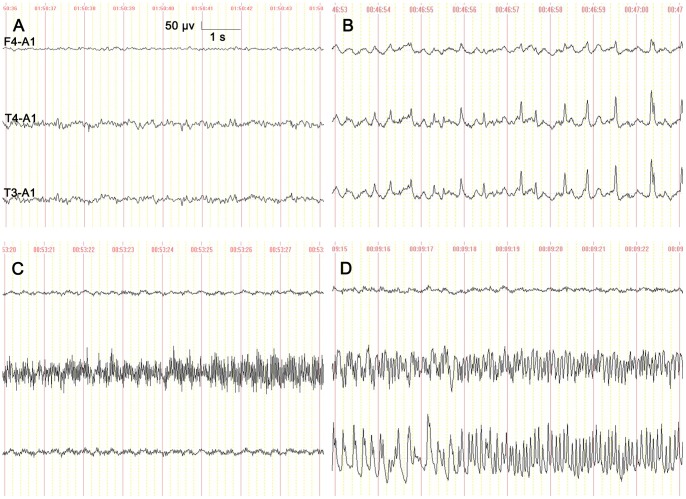
Characterization of electrographic seizure EEG patterns of rats. (A) Normal electrical activity before drug injection; (B) Sporadic interictal sharp wave observed at 4 min after drug injection; (C) Focal EEG seizure with ictal epileptic discharge limited on T4 electrode; and (D) Spreading EEG seizure with ictal epileptic discharge on T4, and T3 or F4 electrode.

**Figure 4:**
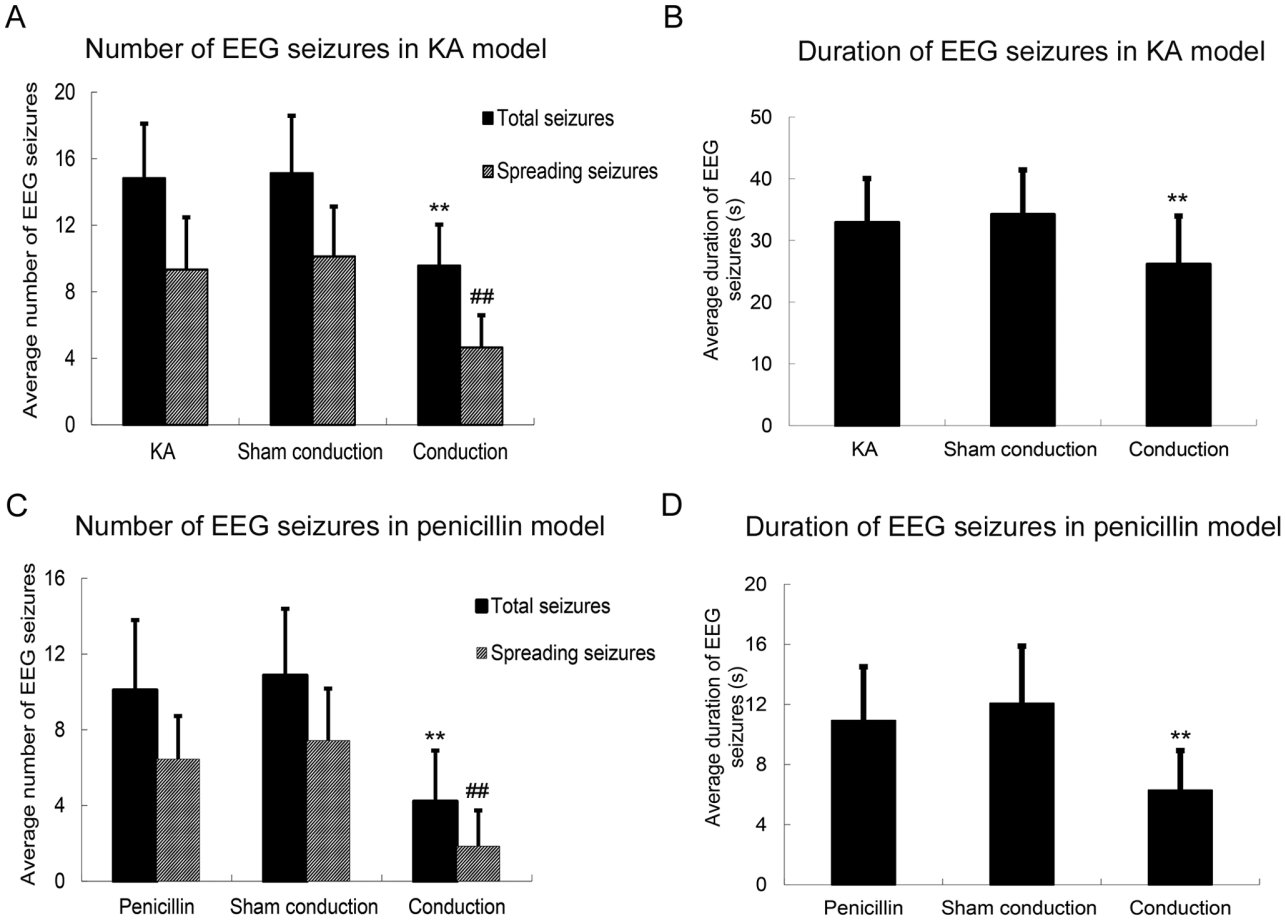
Effect of current shunt on seizures. (A)The average number of total EEG and spreading EEG seizures in the conduction group was significantly lower than that in the KA and sham conduction groups. A 36.7% reduction of total EEG seizures and a 54.1% reduction of spreading EEG seizures were observed in the conduction group compared with sham conduction group. (B) The average duration of EEG seizures in the conduction group (26.13±7.81 s) was significantly lower than that in the KA (32.88±7.16 s) and sham conduction groups (34.17±7.25 s). (C) The average number of total EEG and spreading EEG seizures in the conduction group was significantly lower than that in the penicillin and sham conduction groups. A 61% reduction of total EEG seizures and a 75% reduction of spreading EEG seizures were observed in the conduction group compared with the sham conduction group. (D) The average duration of the EEG seizures in the conduction group (6.29±2.64 s) was significantly lower than that in the penicillin (10.92±3.59 s) and sham conduction groups (12.07±3.81 s). The values are expressed as the means ± standard deviation. **p<0.01, ^##^ *p*<0.01, conduction group versus the KA and sham conduction groups, respectively. One-way ANOVA followed by Bonferroni post hoc test.

Sporadic interictal sharp waves were observed in the 3∼7 min following KA injection, reaching EEG seizure in approximately 15 minutes (Figure 3). We analyzed the EEG seizures during the two hours after conduction electrode implantation. Only one rat in the control group experienced focal EEG seizure once. The number of EEG seizures in the model group was significantly higher than that of the control group (*p*<0.01). For the model group, there was a significant difference in total seizures (F = 23.68, *p* = 0.000) and spreading seizures (F = 26.73, *p* = 0.000) among the three groups. The average number of total EEG seizures in the conduction group was 9.57±2.46, significantly less than 14.83±3.27 in KA group (*p*<0.01) and 15.13±3.45 in sham conduction group (*p*<0.01). There was a 36.7% reduction of total EEG seizures in the conduction group compared with the sham conduction group. Spreading EEG seizure presented in all rats in the KA, sham conduction and conduction groups, and no seizures were observed in the control group. The average number of spreading EEG seizures in the conduction group was 4.65±1.94. There was a 50.2% reduction of spreading seizures in the conduction group compared with the KA group, with 9.33±3.14 (*p*<0.01), and a 54.1% reduction compared with the sham conduction group, with 10.13±2.99 (*p*<0.01). No significant differences were observed between the sham conduction and KA groups (*p*>0.05) (Figure 4A).

#### Average duration of EEG seizures

There was significant difference in the duration of EEG seizures among the three groups after KA injection (F = 7.84, *p* = 0.001). The average duration in the conduction group was 26.13±7.81 s, significantly shorter than the 32.88±7.16 s observed in the KA group (*p = *0.008) and the 34.17±7.25 s observed in the sham conduction group (*p* = 0.001). No difference was observed between the KA and sham conduction groups (*p*>0.05) (Figure 4B).

#### Convulsive behaviors

The control rats, receiving saline instead of KA, did not show any behavioral seizures. A total of 70 rats developed acute seizures after KA injection after awaking from anesthesia. The seizures manifested with head nodding, “wet dog shakes”, tremors, a rigid posture, rearing and falling with forelimbs clonus and generalized tonic-clonic seizure. We analyzed the numbers of behavioral seizures in the 70 rats for 72 h (Table 1). No significant differences were observed between the KA and sham conduction groups (*p*>0.05). At 0∼24 h, a significant reduction of total, minor and serious seizures was observed in the conduction group compared with the KA (*p* = 0.000) and sham conduction groups (*p* = 0.000). A 37.8% reduction of total seizures was observed in the conduction group compared with the sham conduction group and a 42.1% reduction of serious seizures was observed between the two groups. At 25∼48 h, a significant reduction of total (*p* = 0.000) and serious seizures (*p* = 0.000) was observed in the conduction group compared with the sham conduction group; however, minor seizures had no significance (*p*>0.05). No serious seizures were observed in the conduction group. At 49∼72 h, a reduction of total and minor seizures was observed in the conduction group compared with the other two groups (*p*<0.01), and no serious seizures were observed in the conduction rats.

**Table 1:** Frequency of behavioral seizures in TLE model (mean ± SD).

	*0∼24 h*			*25∼48 h*			*49∼72 h*		
	Totalseizure	Minorseizure	Seriousseizure	Totalseizure	Minorseizure	Seriousseizure	Totalseizure	Minorseizure	Seriousseizure
***KA (n = 24)***	49.56±4.31	30.29±2.58	19.27±3.18	4.79±1.12	3.10±0.77	1.69±0.67	2.04±0.75	1.96±0.69	0.04±0.20
***Sham-conduction (n = 23)***	51.17±3.99	31.63±2.83	19.52±3.94	4.87±1.15	3.22±0.67	1.65±0.66	2.21±0.90	2.17±0.83	0.04±0.21
***Conduction(n = 23)***	31.83±3.79**	20.48±3.26**	11.3±2.95**	3.02±0.90**	3.02±0.90	0.00±0.00**	0.78±0.65**	0.78±0.65**	0.00±0.00
***F value***	163.251	107.949	41.494	22.341	0.361	71.739	23.535	24.316	0.490
***P value***	0.000	0.000	0.000	0.000	0.698	0.000	0.000	0.000	0.615

** *p*<0.01, conduction group versus the other two groups during the same period, one-way ANOVA followed by Bonferroni post hoc test.

### Part II: Penicillin Induced Neocortical Epilepsy Model

#### Number of EEG seizures

The first epileptiform discharge was observed at 3∼8 min following penicillin injection, reaching EEG seizure in approximately 17 min. No EEG seizure was observed in the control group. In the model group, there was a significant difference between number of total EEG (F = 16.53, *p* = 0.000) and spreading seizures (F = 22.93, *p* = 0.000) among the three groups. No difference was observed between focal EEG seizures among the three groups (F = 2.12, *p* = 0.134). The average number of total EEG seizures in the conduction group was 4.21±2.69, significantly less than that in the penicillin (10.08±3.71, *p*<0.01) and sham conduction groups (10.86±3.53, *p*<0.01). There was a 61% reduction of total EEG seizures in conduction group compared with sham conduction group. A significant difference was also observed between the numbers of spreading EEG seizures among the three groups (F = 22.93, *p* = 0.000). The number of spreading EEG seizures in the conduction group was 1.86±1.88, significantly lower than that observed in the penicillin (6.46±2.26, *p*<0.01) and sham conduction groups (7.43±2.74, *p*<0.01). A 75% reduction in spreading seizures was observed in the conduction group compared with the sham conduction group. No difference was observed between the penicillin and sham conduction groups (*p*>0.05) (Figure 4C).

#### Average duration of EEG seizures

A significant difference was also observed among the three groups after penicillin injection (F = 7.92, *p* = 0.001). The average duration in the conduction group was 6.29±2.64 s, significantly lower than the 10.92±3.59 s observed in the penicillin group (*p* = 0.016) and 12.07±3.81 s observed in the sham conduction group (*p* = 0.002). No difference was observed between the penicillin and sham conduction groups (*p*>0.05) (Figure 4D).

#### Convulsive behaviors

No behavioral seizures were observed in control rats receiving saline instead of penicillin. A total of 41 rats developed acute seizures after penicillin injection upon awaking from anesthesia. The numbers of behavioral seizures for 72 h are shown in Table 2. No significant differences were observed between the penicillin and sham conduction groups (*p*>0.05). At 0∼24 h, a significant decrease in the numbers of total, minor and serious seizures was observed in the conduction group compared with the penicillin (*p*<0.01) and sham conduction groups (*p*<0.01). A 48.4% reduction of total seizures was observed in the conduction group compared with the sham conduction group and a 52.6% reduction of serious seizure was observed between the two groups. At 25∼48 h, significant decreases of total (*p* = 0.005) and minor seizures (*p* = 0.014) were observed in the conduction group compared with sham conduction group. No serious seizures were observed in the conduction group. At 49∼72 h, a reduction of total seizures was observed in the conduction group compared with the other two groups (*p* = 0.025), and no serious seizures were observed in the conduction rats.

**Table 2:** Frequency of behavioral seizures in neocortical epilepsy model (mean ± SD).

	*0∼24 h*			*25∼48 h*			*49∼72 h*		
	Totalseizure	Minorseizure	Seriousseizure	Totalseizure	Minorseizure	Seriousseizure	Totalseizure	Minorseizure	Seriousseizure
***Penicillin (n = 13)***	16.62±5.88	9.38±3.75	7.23±3.14	2.69±1.80	2.08±1.26	0.62±0.96	1.31±1.10	1.00±1.08	0.15±0.38
***Sham-conduction (n = 14)***	17.29±5.78	9.57±3.65	7.71±3.52	2.43±1.65	2.00±1.24	0.36±0.63	1.29±1.07	0.93±0.83	0.21±0.43
***Conduction(n = 14)***	8.93±3.36**	5.50±2.41**	3.43±2.24**	0.86±0.95*	0.86±0.95*	0.00±0.00	0.29±0.61**	0.29±0.61	0.00±0.00
***F value***	11.401	6.654	8.437	5.985	4.831	3.017	5.190	2.915	1.599
***P value***	0.000	0.003	0.001	0.005	0.014	0.061	0.010	0.066	0.215

* *p*<0.05, **p<0.01, conduction group versus the other two groups during the same period, one-way ANOVA followed by Bonferroni post hoc test.

## Discussion

To our knowledge, this study represents the first demonstration of current shunt therapy in epilepsy. The current mechanisms of anti-epilepsy therapies primarily focus on three aspects [5], [25]–[27]: (1) resecting the epileptogenic zone through resective surgery, stereotactic radiofrequency ablation, and stereotactic radiotherapy; (2) blocking the propagation pathway, such as the corpus callosotomy, and multiple subpial transaction; and (3) decreasing cortex excitability, using drugs, neuromodulation therapy and so on. Thus, the basic idea of all traditional therapies is “inhibition”. In the present study, we proposed the use of a current shunt using a microelectrode with low resistance and superconductivity to control seizures. In contrast to traditional therapies, the basic idea of this mechanism is “conduction” rather than “inhibition”.

Due to lack of similar research, we used two different approaches to confirm the efficacy of current shunt treatment. TLE is the most frequent focal epilepsy and is often resistant to anti-epileptic drugs. After anterior temporal lobectomy, 20% to 30% of the patients experience continuous postoperative seizures, and the outcome of seizure control after deep brain and vagus nerve stimulation is inconsistent. The KA-induced epileptic rat is a widely used temporal lobe epileptic model, which closely resembles the EEG, morphological and biochemical abnormalities of human TLE [19]. Therefore, this rat model was employed in the present study. Because KA subtype receptors are particularly abundant in the CA3 region of the hippocampus, and pyramidal cells in this region present intrinsic kindling attributes, we chose this subfield as the injection site. Moreover, because the Schaeffer pathway connects the CA1 region with the CA3 region, generating a nearly simultaneous epileptic discharge in these regions, we inserted the conduction electrode in the CA1 area.

Besides TLE, neocortex epilepsy is another popular epilepsy in epileptic surgery. Therefore, penicillin-induced focal neocortex epilepsy, a well-known model in experimental studies on epilepsy [28]–[31], was employed in the present study. Penicillin is a gamma-aminobutyric acid A (GABA_A_) receptor antagonist, impairing the function of GABA-mediated inhibitory neurotransmission. Because the inhibition is impaired, recurrent excitatory postsynaptic potentials and the intrinsic bursting of a subpopulation of pyramidal cells results in excessive cell firing in interconnected cortical neurons and highly synchronized activity in the neuronal population [31].

Our research confirmed the efficacy of shunt treatment for epileptic discharge. After conduction electrode implantation, we observed a 36.7% reduction of total EEG seizures and a 50.2% decrease of spreading EEG seizure in the conduction group compared with the sham conduction group in the TLE model, suggesting that current shunt via conduction electrode implantation effectively reduces the generation and propagation of KA-induced seizures in EEG. A similar outcome was also observed in the penicillin-induced neocortical epilepsy model. A 61% reduction of total EEG seizures and a 75% decrease of spreading EEG seizure were observed in the conduction group compared with sham conduction group after penicillin injection. Notably, no significant difference in focal EEG seizures among the three groups was observed, potentially reflecting the fact that some spreading EEG seizures degraded to focal EEG seizures after effective current shunt through the conduction microelectrode, thus the reduction percentage of spreading EEG seizures was higher than that of total EEG seizures, while number of focal seizures was unchanged.

The results of behavioral seizures were consistent with the results of the EEG analysis. In the TLE model, a 37.8% reduction of total behavioral seizures and a 42.1% reduction of serious seizures were observed in the conduction group compared with the sham conduction group. In the neocortical epilepsy model, a 48.4% reduction of total behavioral seizures and a 52.6% reduction of serious seizures were observed in the conduction group compared with the sham conduction group. Minor and serious seizures after conduction electrode implantation were obviously reduced on the first day, and serious seizures disappeared on the second day. Therefore, current shunt reduces ictal seizure discharge generation and inhibits epileptic discharge propagation, greatly reducing the number of serious behavior seizures. We compared the behavioral seizures at 72 h to evaluate the efficacy of conduction for the following reasons: (1) in our preliminary experiment, we observed that behavioral seizures occurred frequently at 6 h after KA injection, subsequently attenuated, followed by a significant decrease in the seizure frequency after 24 h; (2) the rats became irritated after 72 h of continuous monitoring; and (3) reviewing the videos to count each grade of seizure is laborious.

Although, promising outcomes were rendered, this study represents the only research focusing on efficacy and potential; thus, some questions still need clarification. As shown in the results, the seizure control levels are not same between the TLE and neocortex epilepsy models, and the reduction percentages of EEG and behavior seizures in the neocortex epilepsy model were higher than those of the TLE model. These observations might reflect differences in the design of the conduction electrode. The contact area between the epileptogenic zone and patch conduction electrode was much larger than that between the epileptogenic zone and needle conduction electrode, potentially resulting in differences in the seizure reductions in the two epileptic models. This result indicates that a more desirable conduction electrode with optimal size or material should be designed and tested in future research.

In conclusion, the present study confirmed the efficacy of current shunt therapy and presented an alternative minimally invasive approach for TLE and neocortex epilepsy. Current shunt obviously reduces EEG seizures and convulsive behaviors, particularly epileptic discharge propagation and serious behavior seizures in rats with temporal lobe epilepsy and neocortex epilepsy.

## Supporting Information

Video S1 The behavioral convulsions of rats after KA injection.
(WMV)

## References

[1] Banerjee PN, Filippi D, Allen Hauser W (2009) The descriptive epidemiology of epilepsy-a review. Epilepsy Res 85: 31–45. 19369037 10.1016/j.eplepsyres.2009.03.003 PMC2696575

[2] Kwan P, Brodie MJ (2000) Early identification of refractory epilepsy. N Engl J Med 342: 314–319. 10660394 10.1056/NEJM200002033420503

[3] Sander JW (2003) The natural history of epilepsy in the era of new antiepileptic drugs and surgical treatment. Epilepsia 44 (Suppl (1)) 17–20. 10.1046/j.1528-1157.44.s.1.1.x 12558826

[4] DeGiorgio CM, Schachter SC, Handforth A, Salinsky M, Thompson J, et al (2000) Prospective long-term study of vague nerve stimulation for the treatment of refractory seizures. Epilepsia 41: 1195–1200. 10999559 10.1111/j.1528-1157.2000.tb00325.x

[5] Theodore W, Fisher R (2004) Brain stimulation for epilepsy. Lancet Neurol 3: 111–1118. 14747003 10.1016/s1474-4422(03)00664-1

[6] Longo DL (2011) Neurologic disorders: Seizures and Epilepsy. In: Harrison’s principals of internal medicine. The McGraw-Hill Companies. Part 17.

[7] Jan MM, Sadler M, Rahey SR (2010) Electroencephalographic features of temporal lobe epilepsy. Can J Neurol Sci 37: 439–448. 20724250 10.1017/s0317167100010441

[8] Javidan M (2012) Electroencephalography in mesial temporal lobe epilepsy: a review. Epilepsy Res Treat doi: 10.1155/2012/637430. Epub2012 Jun 17. 10.1155/2012/637430 PMC3420622 22957235

[9] Raghavendra S, Nooraine J, Mirsattari SM (2012) Role of electroencephalography in presurgical evaluation of temporal lobe epilepsy. Epilepsy Res Treat doi: 10.1155/2012/204693. Epub 2012 Oct 31. 10.1155/2012/204693 PMC3503287 23198144

[10] Sakamoto AC (2004) Current role of EEG in the presurgical evaluation of temporal lobe epilepsy patients. Suppl Clin Neurophysiol 57: 383–391. 16106637 10.1016/s1567-424x(09)70375-7

[11] Bear MF, Coonors BW, Paradiso MA (2001) Neuroscience: Exploring the Brain. 2nd edition, Lippincott Willianms &Wilkins Inc.

[12] Barz H, Schreiber A, Barz U (2013) Impulses and pressure waves cause excitement and conduction in the nervous system. Med Hypotheses 81: 768–772. 23953966 10.1016/j.mehy.2013.07.049

[13] Vagus Nerve Stimulation Study Group (1995) A randomized controlled trial of chronic vagus nerve stimulation for treatment of medically intractable seizures. Neurology 45: 224–230. 7854516 10.1212/wnl.45.2.224

[14] Benabid AL, Pollak P, Louveau A, Henry S, de Rougemont J (1987) Combined (thalamotomy and stimulation) stereotactic surgery of the VIM thalamic nucleus for bilateral Parkinson disease. Appl Neurophysiol 50: 344–346. 3329873 10.1159/000100803

[15] Benabid AL, Pollak P, Gervason C, Hoffmann D, Gao DM, et al (1991) Long-term suppression of tremor by chronic stimulation of the ventral intermediate thalamic nucleus. Lancet 337: 403–406. 1671433 10.1016/0140-6736(91)91175-t

[16] Gross RE, Lozano AM (2000) Advances in neurostimulation for movement disorders. Neurol Res 22: 247–258. 10769817 10.1080/01616412.2000.11740667

[17] Lim SN, Lee ST, Tsai YT, Chen IA, Tu PH, et al (2007) Electrical stimulation of the anterior nucleus of the thalamus for intractable epilepsy: a long-term follow-up study. Epilepsia 48: 342–347. 17295629 10.1111/j.1528-1167.2006.00898.x

[18] Fisher R, Salanova V, Witt T, Worth R, Henry T, et al (2010) SANTE Study Group. Electrical stimulation of the anterior nucleus of thalamus for treatment of refractory epilepsy. Epilepsia 51: 899–908. 20331461 10.1111/j.1528-1167.2010.02536.x

[19] Boon P, Vonck K, De Herdt V, Van Dycke A, Goethals M, et al (2007) Deep brain stimulation in patients with refractory temporal lobe epilepsy. Epilepsia 48: 1551–1560. 17726798 10.1111/j.1528-1167.2007.01005.x

[20] Hodaie M, Wennberg RA, Dostrovsky JO (2002) Chronic anterior thalamus stimulation for intractable epilepsy. Epilepsia 43: 603–608. 12060019 10.1046/j.1528-1157.2002.26001.x

[21] Trevelyan AJ, Sussillo D, Yuste R (2007) Feedforward inhibition contributes to the control of epileptiform propagation speed. J Neurosci 27: 3383–3387. 17392454 10.1523/JNEUROSCI.0145-07.2007 PMC6672122

[22] Paxinos G, Watson C (1997) The Rat Brain in Stereotaxic Coordinates. San Diego: Academic Press.

[23] Wyckhuys T, Boon P, Raedt R, Van Nieuwenhuyse B, Vonck K, et al (2010) Suppression of hippocampal epileptic seizures in the kainite rat by Poisson distributed stimulation. Epilepsia 51: 2297–2304. 20973781 10.1111/j.1528-1167.2010.02750.x

[24] Racine RJ (1972) Modification of seizure activity by electrical stimulation. Electroencephalogr Clin Neurophysiol 32: 281–294. 4110397 10.1016/0013-4694(72)90177-0

[25] Liang S, Li A, Jiang H, Meng X, Zhao M, et al (2010) Anterior corpus callosotomy in patients with intractable generalized epilepsy and mental retardation. Stereotact Funct Neurosurg 88: 246–252. 20530978 10.1159/000315462

[26] Liang S, Liu T, Li A, Zhao M, Yu X, et al (2010) Long-term follow up of very low-dose LINAC based stereotactic radiotherapy in temporal lobe epilepsy. Epilepsy Res 90: 60–67. 20403679 10.1016/j.eplepsyres.2010.03.008

[27] Leach JP, Abassi H (2013) Modern management of epilepsy. Clin Med 13: 84–86. 10.7861/clinmedicine.13-1-84 PMC5873717 23472502

[28] Makiranta M, Ruohonen J, Suominen K, Niinimaki J, Sonkajarvi E, et al (2005) Bold signal increase preceeds EEG spike activity–a dynamic penicillin induced focal epilepsy in deep anesthesia. Neuroimage 27: 715–724. 16006147 10.1016/j.neuroimage.2005.05.025

[29] Canan S, Ankarali S, Marangoz C (2008) Detailed spectral profile analysis of penicillin-induced epileptiform activity in anesthetized rats. Epilepsy Res 82: 7–14. 18657397 10.1016/j.eplepsyres.2008.06.005

[30] Kortelainen J, Silfverhuth M, Suominen K, Sonkajarvi E, Alahuhta S, et al (2010) Automatic classification of penicillin-induced epileptic EEG spikes. Conf Proc IEEE Eng Med Biol Soc 2010: 6674–6677. 21096740 10.1109/IEMBS.2010.5627154

[31] Silfverhuth MJ, Kortelainen J, Ruohonen J, Suominen K, Niinimäki J, et al (2011) A characteristic time sequence of epileptic activity in EEG during dynamic penicillin-induced focal epilepsy–a preliminary study. Seizure 20: 513–519. 21511498 10.1016/j.seizure.2011.03.006

